# Description of two new *Pristionchus* species from South Korea

**DOI:** 10.2478/jofnem-2024-0032

**Published:** 2024-10-04

**Authors:** Matthias Herrmann, Natsumi Kanzaki, Christian Weiler, Penghieng Theam, Christian Rödelsperger, Ralf Sommer

**Affiliations:** Max Planck Institute for Biology Tübingen, Department of Integrative Evolutionary Biology, Max-Planck Ring 9, 72076 Tübingen, Germany; Kansai Research Center, Forestry and Forest Products Research Institute (FFPRI), 68 Nagaikyutaroh, Momoyama, Fushimi, Kyoto 612-0855, Japan

**Keywords:** Diplogastridae, *Pristionchus*, *hangukensis*, *coreanus*, n. sp., Molecular phylogeny, South Korea

## Abstract

Based on molecular markers, mating experiments, morphological observations and ecological data, two *Pristionchus* species (Nematoda: Diplogastridae) new to science are described. Both were collected from different Scarabaeoid beetles in South Korea, have a gonochoristic mode of reproduction and fall into a sub-clade of the *pacificus* clade. *Pristionchus coreanus* n. sp. does not show a eurystomatous morph under laboratory conditions and might therefore be suitable for the study of gain and loss of polymorphism. *Pristionchus hangukensis* n. sp. is phylogenetically close to Chinese and Japanese species and helps to separate an Asian clade from an American clade.

The nematode genus *Pristionchus* Kreis 1932 gained interest in the scientific community due to the importance of the species *Pristionchus pacificus* in ( 24) as a model organism in eco–evo–devo (22). While original studies focused on comparisons to *Caenorhabditis elegans* (for review see 31), work over the last decade established *P. pacificus* as a genetic, molecular and ecological model organism to study developmental plasticity of its feeding structures (for review see 32). Specifically, most *Pristionchus* species can form two alternative mouth forms, one of which enables predation on other nematodes (19,33). Predation can in principle be cannibalistic and requires sophisticated self-recognition mechanisms to distinguish kin from non-kin at the intra- and interspecies level (34,35). The analysis of such complex organismal traits heavily depends on a rich collection of *Pristionchus* material from nature. To bring more *P. pacificus* strains and additional species of the genus *Pristionchus* into the laboratory, (37,38). However, our sampling efforts throughout Asia are far from being complete and as a result, all of the last sampling expeditions have resulted in the isolation and characterization of species new to science (13). To increase the number of described species as well as our observation range, we conducted a collecting trip to the South Korean peninsula and the island of Jeju in summer 2022.

## Materials and Methods

### Nematode isolation

From June 26^th^ to July 11^th^, 2022, we travelled to South Korea and sampled potentially nematode harboring insects at different locations. Beetles of the Superfamily Scarabaeoidea were collected using light traps (Lepi LED) at several locations in South Korea. Beetles were brought to our laboratory in Germany alive. There, they were dissected and put on individual Petri dishes with NGM agar. Plates were scanned for developing nematodes over two weeks. Single gravid females were transferred to new plates with NGM agar and *E. coli* strain OP50 as food. Initial bacterial contamination was eradicated by bleaching cultures with NaOH&Hydrogenperoxide.

After the establishment of isogenic lines, followed by successful freezing in liquid nitrogen confirmed by thawing tests, these lines got ‘RS’ numbers and database entries were generated.

### Morphological observation

Live adults of *P. hangukensis* n. sp. and *P. coreanus* n. sp. from two 1–2-week-old cultures were used for morphological observations. Because the eurystomatous form was not observed in *P. coreanus* n. sp. using *E. coli* OP50 as food, we attempted to induce this mouth form using *Novosphingobium* sp. as a food source of the species because previous work has shown that this bacterial strain regularly induces the predatory morph (39). Morphological observations and drawings were performed using a microscope (Zeiss Axioskop) equipped with differential interference contrast optics and a drawing tube following the methods of (19)). Light micrographs were taken with a microscope (Zeiss AxioImager Z1) equipped with differential interference contrast optics and a microscopic digital camera (Zeiss Axiocam 506 mono) operated by computer software ZEN (Zeiss). The drawings and micrographs were edited using Photoshop Elements 2020 software (Adobe) to construct figures.

To prepare type material, nematodes were isolated from cultures, rinsed in distilled water to remove bacteria, heat killed at 65°C, fixed in 5% formalin and processed through a glycerol and ethanol series using Seinhorst’s method (9). Then, single specimens were mounted on microscopic slides in glycerol, surrounded by a wax ring and labeled.

### Remark concerning morphometric measurements

We use a multi-dimensional approach for modern description of nematodes, combining molecular, morphological, biological and ecological information to characterize new material as broadly as possible. This is based on our repeated findings that measurements of certain nematode dimensions can change over time as a result to different culture conditions (3,7,10,11,20).

### Molecular characterization, phylogeny and reproductive isolation

A species phylogeny of the complete *Pristionchus* genus was reconstructed as described in (18). In brief, transcriptome libraries were generated from mixed-stage worm cultures and sequenced on an Illumina HiSeq 3000. Raw reads were assembled *de novo* and clustered into orthologous gene families. Protein sequences of more than 2,000 orthologous gene clusters without duplications were further aligned and a maximum likelihood tree was reconstructed based on the concatenated alignment (please see (18) for full details).

### Mating experiments

To examine reproductive isolation of the isolated strains, we always performed crosses with the most closely related strains from our collection. Mating experiments were performed as previously described with some modifications (5). Three virgin females of a strain were tested with 6 males of another strain on a mating plate that contained a small amount of *E. coli* OP50 bacteria. All crosses were performed reciprocally and in quadruplicate. To test for the production of viable F1 hybrids, crosses were set up between all possible pairs and F1 hybrids were tested for their ability to produce F2 progeny. We considered strains to belong to the same species if they produced viable and fertile offspring.

## Results

### Molecular profiles and phylogeny

(Fig. 1).

**Figure 1:**
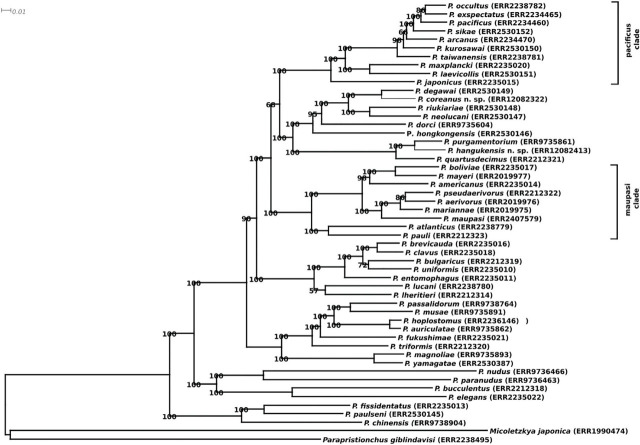
Molecular phylogeny of *Pristionchus* species. The phylogeny shows a maximum likelihood tree that was inferred from a concatenated alignments of more than 1200 orthologous gene clusters. Branch lengths reflect the estimated number of amino acid substitutions per site and branch labels show bootstrap support values (100 pseudoreplicates). The accession numbers next to the species names denote the Run accession in the European nucleotide Archive, where the raw RNA-seq reads were deposited.

### Description

#### Description of common characters

Most *Pristionchus* species, except for some basal clades and fig-associates, are typologically similar to each other and generally distinguished based on stomatal and male tail characters. Therefore, the general morphology is described first as common characters for the two new species, and subsequently the distinctive characters are described for each species.

#### Adult

The body was cylindrical, stout, and the cuticle was thick, with fine annulation and clear longitudinal striations. Lateral field consisted of two lines, only weakly distinguishable from body striation with the presence of deirid. Head was without apparent lips, and with six short and papilliform labial sensillae; four small, papilliform cephalic papillae were present in males. Amphidial apertures located on the lateral sector, slightly dorsally shifted, at the level of margin of cheilo- and gymnostom. Detailed stomatal morphology is described below for each species. The dorsal pharyngeal gland was clearly observed, penetrating dorsal tooth to gland opening. Anterior part of pharynx (=pro and metacorpus) was 1.5 times as long as posterior part (=isthmus and basal bulb); the procorpus very muscular and stout, occupying half to two-thirds of corresponding body diameter; the metacorpus was very muscular, forming a well-developed median bulb; isthmus was narrow, not muscular; basal bulb was glandular. Pharyngo-intestinal junction was clearly observed and well developed. Nerve ring usually surrounded middle region of isthmus; excretory pore was not conspicuous and ventrally located at level of isthmus to pharyngo-intestinal junction, the excretory duct extended anteriad and reflexed back to position of the pore. Deirid was observed laterally, located from the slightly anterior to pharyngo-intestinal junction to a half body diameter posterior to the junction. Hemizonid was not observed. Lateral glands, small pores connected to secretory cell, were present and observed on the lateral body surface, with positions inconsistent among individuals, numbering 5 to 8 for males and 9 to 13 for females. Postdeirid was at anterior part of *vas deferens* in male and the posterior part of posterior gonad in female, on the second dorsally neighboring striation to the lateral field.

#### Male

Body was ventrally arcuate, strongly ventrally curved at tail region when killed by heat. Testis single, ventrally located and anterior part reflexed to right side. Spermatogonia arranged in three to five rows in reflexed part, then well-developed spermatocytes arranged as three to four rows in anterior two-thirds of the main branch, then mature amoeboid spermatids arranged in multiple rows in remaining, proximal part of gonad; *vas deferens* not clearly separated from other parts of gonad. Three (two subventral and one dorsal) cloacal gland cells were observed at distal end of testis and intestine. Spicules paired, separate; spicules smoothly curved in ventral view, adjacent to each other for the distal third of their length, each smoothly tapering to pointed distal end; spicule in lateral view was smoothly ventrally arcuate, giving spicule about 100° curvature, oval manubrium was present at anterior end, lamina/calomus complex (blade) was clearly expanded slightly posterior to manubrium (*ca* 1/4 of blade length from anterior) and was then smoothly tapering to the pointed distal end. Gubernaculum conspicuous, about one-third of the spicule length, was broad anteriorly such that the dorsal wall was slightly recurved with dorsal and ventral walls separate at a 50 to 60° angle at the posterior end; the dorsal side of the gubernaculum possessing a single, membranous, anteriorly directed process and lateral pair of more sclerotized, anteriorly and obliquely ventrally directed processes. In the lateral view, the anterior half of gubernaculum was with two serial curves separated by an anteriorly and obliquely ventrally directed process, with anterior terminal curvature highly concave and almost closed, and with deep posterior curvature being one-third of the gubernaculum length; the posterior half was forming a tube-like process enveloping spicules. There was a thick cuticle around the tail region, falsely appearing as a narrow leptoderan bursa in the ventral view. Cloacal opening (co) was slit-like in the ventral view; one small, ventral, single genital papilla (vs) was on the anterior cloacal lip; nine pairs of genital papillae (v1–v7, ad, pd) and a pair of phasmids (ph) were present, and three precloacal and six postcloacal pairs. Within those pairs, three distal ventral pairs (v5, v6, and v7) and a dorsal pair (pd) were close to each other around the posterior end of tail (just anterior to the root of the spike). Anterior five pairs of papillae (v1–4 and ad) were almost equal in size and rather large and conspicuous, v7 and pd papillae were obviously smaller than the anterior five pairs, v5 and v6 were very small and sometimes difficult to observe with a light microscope. There were anterior two pairs of the ventral triplet papillae (v5 and v6) papilliform and they were borne from the socket-like base and were v7 simple or typical thorn-like in shape. The tip of v6 papillae split into two small papilla-like projections. Detailed arrangement of papillae and phasmid will be described for each species below. Tail conical, with a long spike, was about two to three cloacal body diameters (CDB) long; the bursa or bursal flap was absent.

#### Female

The body was relaxed or slightly ventrally arcuate when killed by heat. Gonad didelphic, amphidelphic; each gonadal system arranged from vulva/vagina as uterus, oviduct, and ovary. Anterior gonads right of intestine with uterus and oviduct extending ventrally and anteriorly on right of intestine and with a totally reflexed (=antidromous reflexion) ovary extending dorsally on left of intestine; oocytes mostly arranged in three to four rows in distal two-thirds of ovary and in double or single rows in rest of the ovary, with the distal tips of each ovary reaching the oviduct of the opposite gonad branch; the anterior end of the oviduct (=junction tissue between ovary and oviduct) consisted of rounded cells; anterior part of oviduct consisted of rounded cells, forming a simple tube; middle part of the oviduct served as spermatheca, consisting of roundish and relatively large cells. Eggs in single to multiple-cell stage or even further developed at the posterior part of the oviduct (=uterus) in young females were being composed of squared or angular cells long enough to contain one well developed oocyte. *Receptaculum seminis* was not observed, i.e., the organ is not independent, and a part of oviduct/uterus works as the organ; vaginal glands were present but obscure; vagina was perpendicular to the body’s surface, surrounded by sclerotized tissue; vulva was slightly protuberant in lateral view, pore-like in ventral view; rectum about one anal body diameter long, the intestine/rectum junction was surrounded by a well-developed sphincter muscle; three anal glands (two subventral and one dorsal) were present but not obvious; anus in the form of dome-shaped slit, posterior anal lip slightly protuberant; phasmid about one to two anal body diameters posterior to anus; tail was long, smoothly tapered, with the distal end variable from filiform to long and conical.

**Figure 2:**
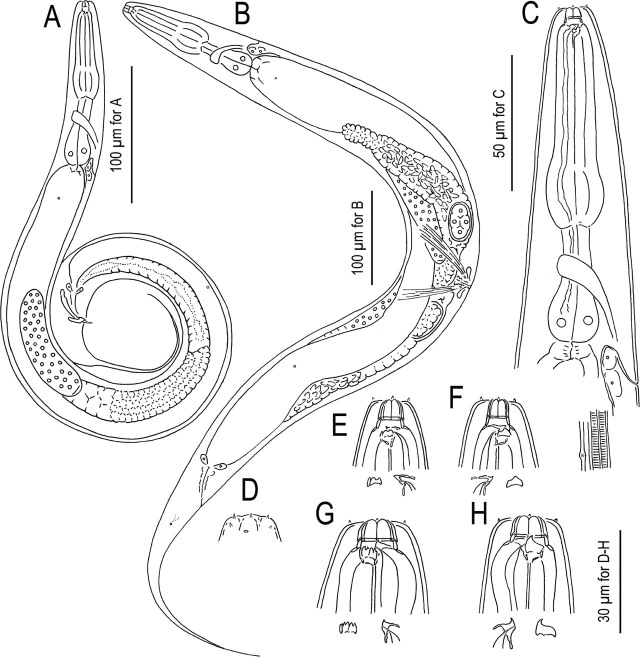
*Pristionchus hangukensis* n. sp. A: Male; B: Female; C: Anterior region; D: Relative position of secretory-excretory pore, deirid and one of lateral glands; E: Surface of anterior end of adult male in right lateral view showing the relative position of labial sensilla, cephalic sensilla and amphid; E, F: Male stenostomatous form in left (E) and right (F) lateral views; G, H: Male eurystomatous form in left (G) and right (H) lateral views. Tooth and denticules are separately drawn under whole head region in E–H.

**Figure 3:**
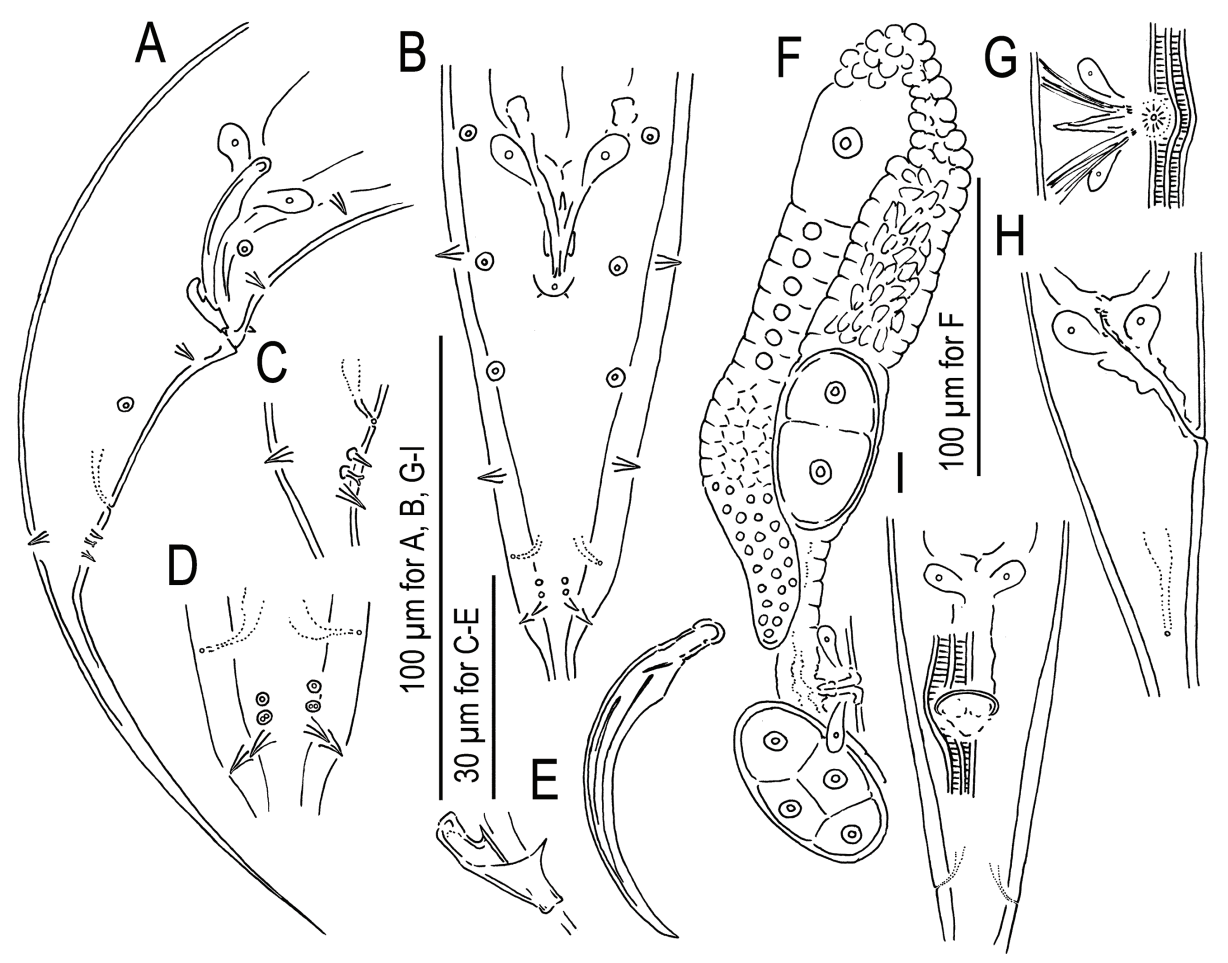
*Pristionchus hangukensis* n. sp. A, B: Whole male tail in right lateral (A) and ventral (B) views; C, D: Close-up of posterior four pairs of genital papillae and phasmid in right lateral (C) and ventral (D) views; E, F: Spicule and gubernaculum of two different individuals in right lateral view; G: Ventral view of female vulval region; H, I: Anal region of female in left lateral (H) and ventral (I) views.

**Figure 4:**
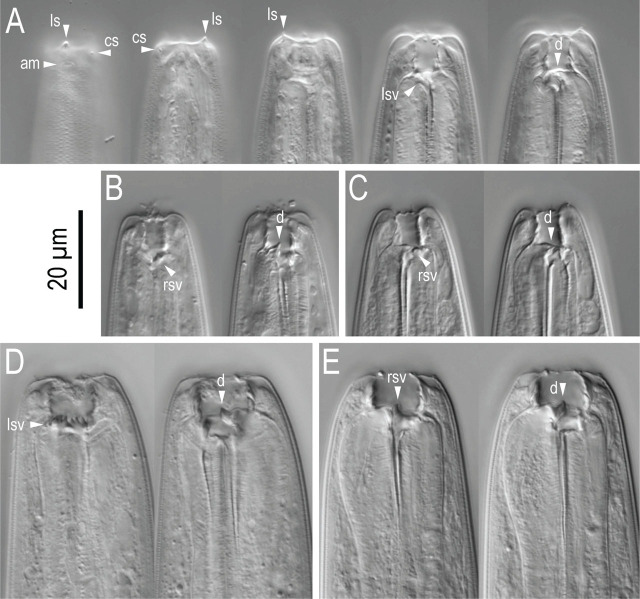
Stomatal region of *Pristionchus hangukensis* n. sp. showing variation among individuals. A: Surface and stoma of male in left lateral view in five different focal planes showing the relative position of labial sensilla, cephalic sensilla, amphid, left subventral ridge and dorsal tooth; B, C: Male (B) and female (C) stenostomatous form in right lateral views in two different focal planes; D, E: Female eurystomatous form in left (D) and right (E) lateral views in two different focal planes. Abbreviations are as follows: am = amphid; cs = cephalic sensilla; d = dorsal tooth; ls = labial sensilla; lsv = left subventral ridge; rsv = right subventral ridge or tooth.

**Figure 5:**
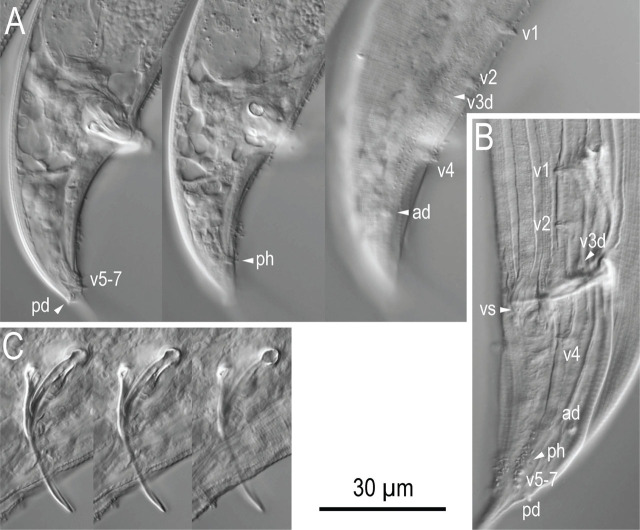
Male tail characters of *Pristionchus hangukensis* n. sp. A: Right lateral view of whole tail in three different focal planes; B: Left subventral view of whole tail; C: Spicule and gubernaculum in right lateral view in three focal planes. Abbreviations are as follows: ad, pd and v + number = genital papillae, where ending with ‘d’ suggests that the papillae are directed laterally or subdorsally; ph = phasmid; vs = ventral single papilla.

^*^Etymology

Pristionchus hangukensis^*^ n. sp. (Figs. 2–5)

So far, the species has only been found in South Korea. Hanguk is the traditional and informal name for South Korea.

#### Measurements

See Table 1.

**Table 1:** Morphological measurements of the two new species.

**Character**	***Pristionchus coreanus* n.sp. RS6268**		***Pristionchus hangukensis* n.sp. RS6291**	

	**stenostomatous male**	**stenostomatous female**	**stenostomatous male**	**stenostomatous female**
n	10	10	10	10
L	1002 ± 94.3 (872 – 1158)	1448 ± 126.0 (1312 – 1661)	928 ± 54.8 (858 – 1001)	1336 ± 90.2 (1248 – 1542)
L’	851 ± 93.3 (721 – 1025)	1211 ± 120.4 (1067 – 1442)	764 ± 41.0 (685 – 820)	1078 ± 76.5 (993 – 1249)
a	13 ± 2.2 (9.1 – 16)	12 ± 1.5 (11 – 16)	13 ± 1.3 (11 – 16)	13 ± 0.7 (11 – 14)
b	6.0 ± 0.4 (5.5 – 6.7)	7.6 ± 0.5 (6.8 – 8.4)	7.0 ± 0.4 (6.5 – 7.6)	9.0 ± 0.7 (8.0 – 10.0)
c	6.7 ± 0.9 (5.7 – 8.5)	6.1 ± 0.5 (5.4 – 7.1)	5.8 ± 0.4 (5.3 – 6.6)	5.3 ± 0.5 (4.9 – 6.3)
c’	3.4 ± 0.5 (2.5 – 4.0)	4.4 ± 0.6 (3.8 – 5.5)	4.4 ± 0.5 (3.9 – 5.3)	6.4 ± 0.6 (5.5 – 7.7)
Ant. stoma length (cheilo- + gymnostom)	5.1 ± 0.8 (4.0 – 6.7)	5.7 ± 1.4 (4.0 – 7.8)	6.9 ± 1.1 (5.0 – 9.3)	7.7 ± 0.7 (6.7 – 9.1)
Total stoma length	11 ± 1.0 (9.3 – 12)	12 ± 1.1 (11 – 14)	10 ± 1.1 (8.3 – 11.6)	13 ± 1.0 (11 – 14)
Stoma width	5.8 ± 0.4 (5.3 – 6.5)	7.5 ± 0.9 (6.0 – 8.7)	4.9 ± 0.9 (3.6 – 6.5)	5.4 ± 0.9 (4.3 – 7.5)
Ant. pharynx (pro + metacorpus)	95 ± 7.6 (83 – 111)	109 ± 8.2 (99 – 130)	69 ± 5.6 (63 – 82)	79 ± 6.0 (72 – 89)
Post. pharynx (isthmus + basal bulb)	67 ± 7.0 (56 – 78)	77 ± 5.1 (69 – 87)	57 ± 2.9 (54 – 61)	61 ± 3.6 (55 – 66)
Total pharynx length	162 ± 13.6 (142 – 183)	186 ± 12.1 (176 – 217)	126 ± 7.6 (120 – 143)	140 ± 7.4 (131 – 152)
Ant./total pharynx %	59 ± 1.7 (57 – 62)	58 ± 1.5 (56 – 62)	55 ± 1.6 (52 – 57)	57 ± 2.2 (54 – 60)
Median bulb diameter	27 ± 2.5 (24 – 32)	35 ± 2.9 (31 – 40)	23 ± 1.2 (21 – 24)	27 ± 2.0 (25 – 31)
Terminal bulb diameter	28 ± 3.9 (22 – 36)	36 ± 4.9 (31 – 46)	22 ± 1.1 (20 – 23)	27 ± 2.3 (24 – 33)
Ant. end to cardia	167 ± 13.2 (151 – 191)	192 ± 8.8 (185 – 212)	132 ± 8.4 (123 – 148)	148 ± 7.5 (139 – 160)
Ant. end to S-E pore	149 ± 15.5 (125 – 170)	195 ± 15.5 (171 – 214)	114 ± 11.3 (99 – 139)	153 ± 12.0 (137 – 172)
Ant. end to nerve ring	118 ± 9.2 (109 – 133)	136 ± 8.9 (124 – 155)	97 ± 6.2 (90 – 108)	111 ± 6.5 (104 – 124)
Testis length	703 ± 83.8 (571 – 827)	-	630 ± 59.4 (529 – 718)	-
Ant. end to vulva distance	-	691 ± 66.1 (600 – 821)	-	608 ± 40.2 (563 – 703)
Vulva to anus distance	-	525 ± 60.0 (454 – 628)	-	473 ± 37.3 (420 – 543)
T or V	70 ± 4.3 (66 – 80)	48 ± 1.5 (46 – 50)	68 ± 6.7 (53 – 77)	46 ± 1.1 (43 – 47)
Max. body diameter	78 ± 19.1 (63 – 127)	121 ± 16.4 (91 – 141)	71 ± 5.1 (64 – 81)	105 ± 8.1 (93 – 115)
Cloacal or anal body diameter	45 ± 5.3 (40 – 54)	54 ± 5.3 (43 – 63)	36 ± 2.2 (33 – 39)	39 ± 3.5 (33 – 45)
Tail length	149 ± 10.8 (129 – 161)	237 ± 23.7 (208 – 296)	159 ± 13.9 (132 – 178)	252 ± 27.0 (204 – 297)
Spicule curve	50 ± 4.3 (40 – 54)	-	46 ± 2.3 (43 – 50)	-
Spicule chord	42 ± 2.9 (36 – 47)	-	38 ± 2.6 (35 – 43)	-
Gubernaculum length	18 ± 2.4 (13 – 21)	-	18 ± 1.6 (16–22)	-

#### Stenostomatous form

Cheilostom consisted of six per- and interradial plates. Incision between plates was not always easily distinguished. Anterior end of each plate was rounded and elongated to project from the stomal opening forming a small flap. Gymnostom was short, cuticular ring-like anterior end overlapping the cheilostom internally. Stegostom separated into three subsections: promeso, meta and telostegostom. Pro-mesostegostom formed a weakly cuticularized indistinctive ring internally overlapping with gymnostom to connect the gymnostom and metastegostom. Metastegostom bore the conspicuous and movable triangular or flint-shaped dorsal tooth with a strongly sclerotized surface giving an appearance of an inverted V-shape in light microscopy in lateral view; the left subventral ridge was with three, minute adventitious blunt denticles on a plate, most ventral denticles were often masked by the remaining two in lateral view; pointed or blunt right subventral ridges were often with a distinct distal adventitious denticle. Because of the size difference between male and females, i.e., females generally have a larger stoma than males, tooth and denticles are larger (wider) in females than males. Telostegostom weakly sclerotized cup-like cavity connecting the stoma and pharynx.

#### Eurystomatous form

Cheilostom was divided into six well-distinguished per- and interradial plates. Anterior end of each plate was rounded and elongated to protrude from stomal opening and form a small flap. Gymnostom was with a thick cuticle, forming a short, ring-like tube being thicker posteriorly; and the anterior end of the gymnostom internally overlapped the posterior end of the cheilostomatal plates. Stegostom separated into three subsections: pro-meso, meta and telostegostom. Pro-mesostegostom formed a weakly cuticularized indistinctive ring internally overlapping with the gymnostom to connect the gymnostom and metastegostom. Metastegostom bore a large claw-like dorsal tooth, and large, claw-like right subventral tooth sometimes with a small indistinct extra peak on its ventral side. Left subventral sector of metastegostom bore three triangular ridges; the tip of each ridge sometimes split into two or more fine tips, and the shape varied among individuals. As in stenostomatous form, the teeth and denticles are generally larger in females than in males. The dorsal tooth and right subventral tooth were movable. Movement was not observed in the left subventral denticles. Telostegostom weakly sclerotized a cup-like cavity connecting the stoma and pharynx.

#### Male

Nine pairs of genital papillae and a pair of phasmid were present with an arrangement <v1, v2, v3d / v4, ad, ph, (v5, v6, v7, pd)>, where v1 located about 1–1.5 cloacal body diam. (CBD) anterior to co; v2 about 0.5 CBD anterior to co; v3d slightly (less than 1/4 CBD) posterior to v2; v4 about 1/4 CBD posterior to co, i.e., v2, v3d, co and v4 were close to each other; ad about 1 CBD posterior to co; ph immediately anterior to v5; v5–v7 forming triplet, immediately anterior to the root of tail spike; and pd around the level of or slightly posterior to v7. The distance between v1 and v2 is generally shorter than that between v2 and v4. The papillae v1, v2, v4 and ph subventral, v3d and ad lateral, v5–7 ventral, pd subdorsal in the male tail. Within nine paired papillae, v1–v4 and ad were the same size, v7 and pd were smaller than former four pairs, and v5 and v6 were smaller than v7 and ad. All nine paired genital papillae papilliform, but v5 and v6 are baring from a socket-like base, and the tip of v6 laterally split into two small tips. Tail conoid with a long spike which occupies more than 60% of the whole tail length. Bursa or bursal flap was absent.

#### Female

As described for common character.

#### Type host and locality

*Pristionchus hangukensis* RS6291 was collected from a specimen of *Dorcus titanus castanicolor* Motschulski 1861 (Coleoptera: Lucanidae) caught at a light trap at N33°15.498′E126°21.456′ on July 3^rd^, 2022, while eating a picknick style dinner with our Korean colleagues near “the road to contemplation” (Chusa or Kim Jeong-Hui). RS6271, another strain of this species was collected at the same location from an *Anomala* sp. (Coleoptera: Rutelidae).

### Type designation and deposition

The type material was obtained from a 2-week-old culture. The samples included a holotype male (collection ID), four paratype males (collection ID) and five paratype females (collection ID) deposited at the Natural History Museum Karlsruhe, Germany, and five paratype males (collection ID) and five paratype females (collection ID) deposited at the Swedish Museum of Natural History, Stockholm, Sweden. In addition to the type material, mass-fixed materials (fixed in TAF or processed in dehydrated glycerin) were deposited at the Max Planck Institute for BiologyTübingen, Germany.

As the strain RS6291 is also frozen in liquid nitrogen at the MPI for Biology Tübingen, it can be provided upon request as a living culture. urn:lsid:zoobank.org:act:960C79FF-D1F0-440BB7B4-36539ECA5F86.

#### Differential diagnosis

*Pristionchus hangukensis* n. sp. is characterized by its stomatal morphology, i.e., flint-shaped dorsal tooth with or without the anteriorly directed curvature at the tip, the left subventral ridge with three blunt denticles and right subventral ridge with a small pointed or blunt denticle of the stenostomatous form; claw-like right subventral stegostomal tooth sometimes with an indistinctive extra peak, and three left subventral ridges each with two or more pointed tips in eurystomatous form, the arrangement of male genital papillae, <v1, v2, v3d / v4, ad, ph, (v5, v6, v7, pd)> where v2, v3d, co and v4 were close to each other, and ph and posterior four paired papillae formed a cluster, and distinctive and long male tail spike. *Pristionchus hangukensis* n. sp. belongs to a clade intermediate between *maupasi* and *pacificus* groups (Fig. 1), which was previously treated as a part of the *maupasi* group (13). Within the clade, *P. hangukensis* n. sp. shares its laterally directed third paired papillae with *P. hongkongensis* Kanzaki, Herrmann, Yoshida, Weiler, Rödelsperger & Sommer, 2018, *P. dorci* (13), *P. purgamentorium* (13) and *P. quartusdecimus* (11). *Pristionchus hangukensis* n. sp. is readily distinguished from *P. hongkongensis* by its whole stomatal shape of the eurystomatous form, regular *pacificus* group-like barrel-shaped stoma *vs* extremely well-developed wide barrel-shaped stoma similar to that of the *triformis* group (12). *Pristionchus hangukensis* n. sp. is distinguished from *P. dorci* by the stomatal elements in the eurystomatous form, i.e., each of the three left subventral ridges with two or more tips *vs* one or two tips, and right subventral tooth with *vs* without indistinctive peak on the ventral side (13). *Pristionchus hangukensis* n. sp. is phylogenetically close to *P. purgamentorium* and *P. quartusdecimus*. However, the new species can be distinguished from *P. purgamentorium* by the right subventral ridge of stenostomatous form with single *vs* multiple peak(s), and from *P. quartusdecimus* by the left subventral ridges of the eurystomatous form, each split into two or more vs one or two small denticles (11,13).

In mating tests, *P. hangukensis* RS6291 was crossed with the *P. purgamentorium* strain RS6138 and also with strain RS6271. Crosses with *P. purgamentorium* RS6138 did not result in offspring while crosses between the strains RS6271 and RS6291 resulted in fertile offspring. Therefore, we consider both RS6291 and RS6271 to be members of one species, described here as *P. hangukensis* n. sp.

The 1616bp fragment of the 18S-SSU (GenBank accession) matches to 99% (1608/1614) with *Pristionchus quartusdecimus* and to 99% (1586/1589) with *P. purgamentorium*.

#### Remarks

Phylogenetically, *P. hangukensis* n. sp. belongs to either an intermediate clade between the *maupasi* and *pacificus* groups, or an independent small clade consisting of *P. hangukensis* n. sp., *P. purgamentorium* and *P. quartusdecimus* (Fig. 1). Further sampling may yield additional phylogenetically close relatives to expand this clade. These three species share a characteristic arrangement of male genital papillae, where the third pair is directed laterally (v3d). As previously noted, the character is found at the basal position of several subclades in the genus, and seemingly the same transition from v3d to v2d occurred independently several times. Further biological examination of these species may reveal the evolutionary and ecological significance of the arrangement of the genital papillae.

**Figure 6:**
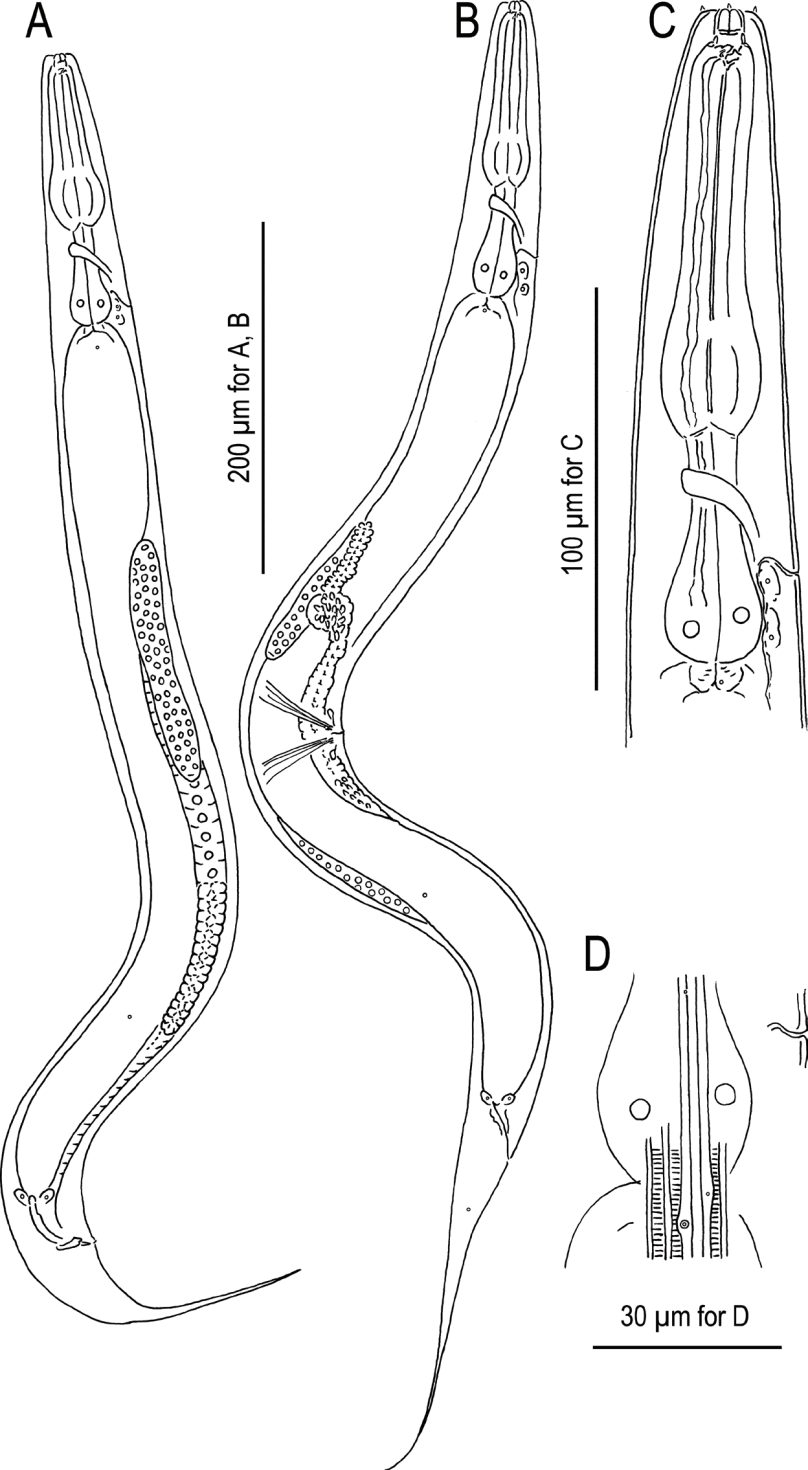
*Pristionchus coreanus* n. sp. A: Male; B: Female; C: Anterior region; D: Relative position of secretory-excretory pore, deirid, and two lateral glands on the lateral field.

**Figure 7:**
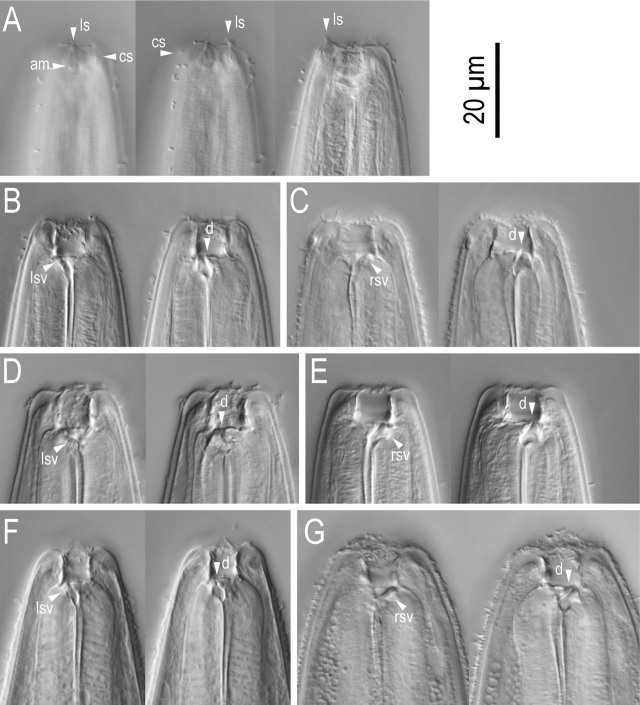
Stomatal region of *Pristionchus coreanus* n. sp. showing variation among individuals. A: Surface of anterior end of adult male in right lateral view showing the relative position of labial sensilla, cephalic sensilla and amphid; B, C: Male stenostomatous form starved culture in left (B) and right (C) lateral views; D, E: Female stenostomatous form from starved culture in left (D) and right (E) lateral views; F, G: Female stenostomatous form from well-fed culture in left (F) and right (G) lateral views. Tooth and denticules are separately drawn under whole head region in B–G.

**Figure 8:**
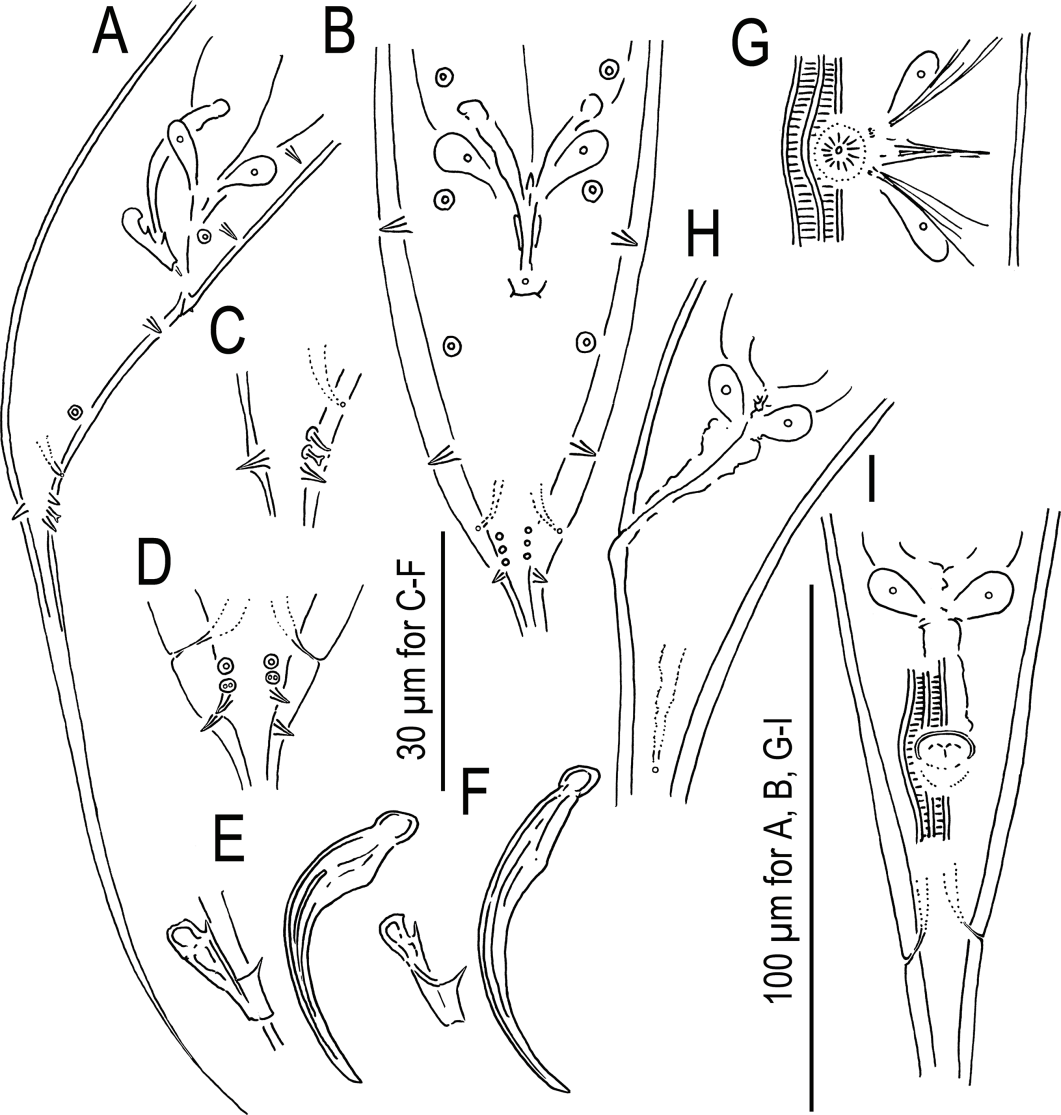
*Pristionchus coreanus* n. sp. A, B: Whole male tail in right lateral (A) and ventral (B) views; C, D: Close-up of posterior four pairs of genital papillae and phasmid in right lateral (C) and ventral (D) views; E: Spicule and gubernaculum in right lateral view; F: Anterior gonad of mature female in right lateral view; G: Ventral view of female vulval region; H, I: Anal region of female in right lateral (H) and ventral (I) views.

**Figure 9:**
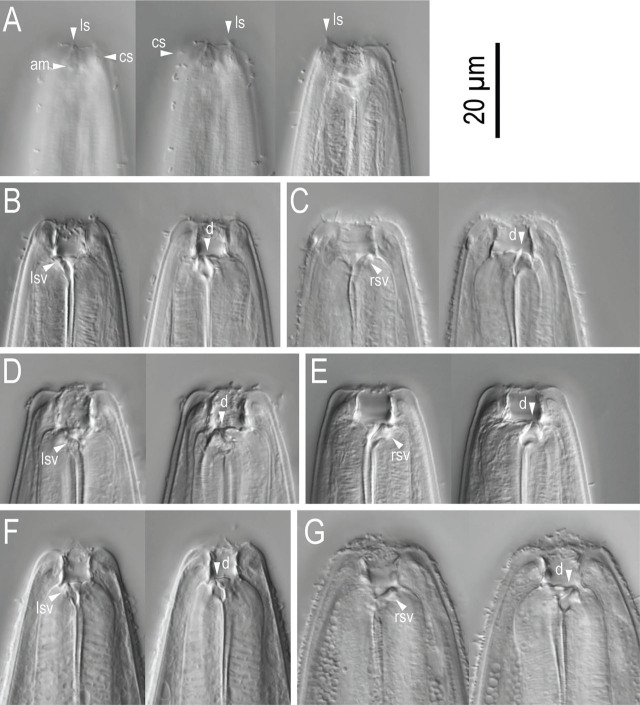
Stomatal region of stenostomatous form of *Pristionchus coreanus* n. sp. showing variation among individuals. A: Surface and stoma of male in left lateral view in three different focal planes showing the relative position of labial sensilla, cephalic sensilla and amphid; B: Left lateral view of male from well-fed culture in two different focal planes; C: Right lateral view of male from starved culture in two different focal planes; D: Left lateral view of female from starved culture in two different focal planes; E: Right lateral view of female from starved culture in two different focal planes; F: Left lateral view of female from well-fed culture in two different focal planes; G: Right lateral view of female from well-fed culture in two different focal planes. Abbreviations are as follows: am = amphid; cs = cephalic sensilla; d = dorsal tooth; ls = labial sensilla; lsv = left subventral ridge; rsv = right subventral ridge or tooth.

**Figure 10:**
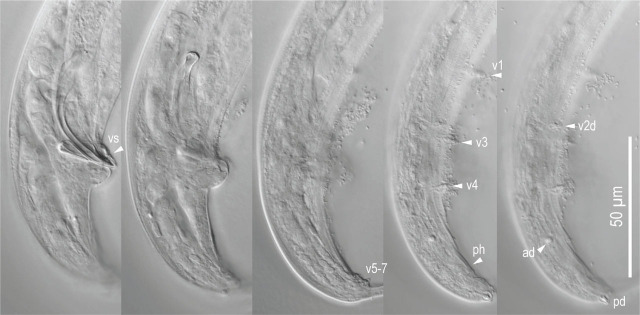
Male tail characters of *Pristionchus coreanus* n. sp. Right lateral view of whole male tail in five different focal planes. Abbreviations are as follows: ad, pd and v + number = genital papillae where ending with ‘d’ suggests that the papillae are directed laterally or subdorsally; ph = phasmid; vs = ventral single papilla.

^*^ *Etymology*

Pristionchus coreanus^*^ n. sp. (Figs. 6–10)

The species is named after the country of origin.

#### Measurements

See Table 1.

#### Stenostomatous form

Cheilostom consisted of six per- and interradial plates. Incision between plates was not always easily distinguished. The anterior end of each plate was rounded and elongated to project from stomal opening and form a small flap. Gymnostom was short, with a cuticular ring-like anterior end overlapping the cheilostom internally. Stegostom was separated into three subsections: pro-meso, meta and telostegostom. Pro-mesostegostom formed a weakly cuticularized indistinctive ring internally overlapping with the gymnostom to connect the gymnostom and metastegostom. Metastegostom bore the conspicuous and movable triangular dorsal tooth with strongly a sclerotized surface giving an appearance of an inverted V-shape in light microscopy in lateral view, the anterior end of the tooth anteriorly curved to form a somewhat claw-like shape; the left subventral ridge was with three adventitious blunt denticles on a plate, most ventral denticles were often masked by the remaining two in lateral view; the pointed or blunt right subventral ridge was often with a distinct distal adventitious denticle. Because of the size difference between males and females, i.e., females generally have a larger stoma than males, tooth and denticles are larger (wider) in females than in males. Whole length-width ratio of the stoma varies among individuals, and relatively small individuals from starved culture tend to have wider stoma. Telostegostom weakly sclerotized a cup-like cavity connecting stoma and pharynx.

#### Eurystomatous form

Not found in regular and induced cultures.

#### Male

Nine pairs of genital papillae and a pair of phasmid were present with an arrangement <v1, (v2d, v3) / v4, ad, ph, (v5, v6, v7, pd)>, where v1 located about 1–1.5 cloacal body diam. (CBD) anterior to co; v2d less than 1/2 CBD anterior to co; v3 slightly (less than 1/4 CBD) posterior to v2; v4 1/4-1/2 CBD posterior to co, i.e., v2, v3d, co and v4 were close to each other; ad about 1 CBD posterior to co; ph immediately anterior to v5; v5–v7 forming triplet, immediately anterior to the root of tail spike; and pd was around the level of triplet, i.e., the v5–v7 and pd were overlapping each other. The distance between v1 and v2 is generally longer than that between v2 and v4. The papillae v1, v2, v4 and ph subventral, v3d and ad lateral, v5–7 ventral, pd subdorsal in the male tail. Within nine paired papillae, v1–v4 and ad were the same size, v7 and pd were smaller than former four pairs, and v5 and v6 were smaller than v7 and ad. All nine paired genital papillae papilliform, but v5 and v6 are baring from the socket-like base, and the tip of v6 laterally split into two small tips. Tail conoid was with a long spike which occupied more than half of the whole tail length. Bursa or bursal flap was absent.

#### Female

As described for common character.

#### Type host and locality

*Pristionchus coreanus* n. sp., type strain RS6268 was isolated from *Lasiopsis sahlbergi* (16), (Coleoptera: Melolonthidae) collected close to the Georin Saseum Observatory bus stop (N33°18.446′E126°27.284′) on July 2^nd^, 2022. The strain RS6267 originated from the same beetle specimen.

#### Type designation and deposition

The type material was obtained from a 2-week-old culture. The samples included a holotype male (collection ID), four paratype males (collection ID) and five paratype females (collection ID) were deposited at the Natural History Museum Karlsruhe, Germany, and five paratype males (collection ID) and five paratype females (collection ID) were deposited at the Swedish Museum of Natural History, Stockholm, Sweden. In addition to the type material, mass-fixed materials (fixed in TAF or processed in dehydrated glycerin) were deposited at the Max Planck Institute for Biology Tübingen, Germany.

As the strain RS6268 is also frozen in liquid nitrogen at the MPI for Biology Tübingen, it can be provided upon request as a living culture. urn:lsid:zoobank.org:act:79BB22D3-2D25-401B-803B-69594562F3CF.

#### Differential diagnosis

*Pristionchus coreanus* n. sp. is characterized by its stomatal morphology of the stenostomatous form, i.e., the left subventral ridge composed by three bump-like denticles, right subventral ridge with a single or blunt peak, and a flint-shaped dorsal tooth with conspicuous anterior curvature, and the arrangement of male genital papillae, <v1, (v2d, v3) / v4, ad, ph, (v5, v6, v7, pd)>. In addition, rather high intraspecific variation of stomatal morphology and the absence of the eurystomatous form can be noted as its diagnostic characters. *Pristionchus coreanus* n. sp. belongs to a clade intermediate between *maupasi* and *pacificus* groups (Fig. 1), which was previously treated as a part of the *maupasi* group (13). Within the clade, *P. coreanus* n. sp. shares the arrangement of male genital papillae, where the second pair is directed laterally (v2d), with *P. degawai* (12) *P. riukiariae* (12) and *P. neolucani* (12). However, *P. coreanus* n. sp. is distinguished from *P. degawai* by the arrangement of genital papillae, v2d and v3 were close to each other by clearly separate vs very close to each other and sometimes overlapping; from *P. riukiariae* by the right subventral ridge of the stenostomatous form possessed a single pointed or blunt peak *vs* two peaks; and from *P. neolucani* by the arrangement of male genital papillae, where v2d and v3 were close to each other in both species, but these two pairs were closer in *P. neolucani*, i.e., these two pairs were always separable in *P. coreanus* n. sp., but sometimes very close and overlapping in *P. neolucani* (12). In addition to these characters, the absence (or difficulty in occurrence) of the eurystomatous form and high intraspecific variation in the width-depth ratio of stoma in the stenostomatous form can differentiate new species from these three close relatives.

To examine reproductive isolation of *P. coreanus* with related species, we crossed the type strain RS6268 with *Pristionchus degawai* strain RS5999, which is the closest relative. As a control, we crossed RS6268 with RS6267. No viable offspring was observed in the crosses with *P. degawai* RS5999. In contrast, RS6268 mates with RS6267 in both ways and crosses always resulted in fertile offspring.

The 1619bp fragment of the 18S-SSU (GenBank accession) matches to 99% (1601/1610) with *Pristionchus neolucani* and to 99% (1596/1602) with *P. degawai*.

## Discussion

This study adds two new species to the genus *Pristionchus* and indicates further variation in the number of mouth forms. While the majority of *Pristionchus* species and many other diplogastrid nematodes have two alternative mouth forms, several independent evolutionary transitions have been observed in this family. The evolution of a third morph is well documented in *Pristionchus* (i.e., *P. triformis*, 11) and was recently also described in the distant relative *Allodiplogaster sudhausi* (28). Fig-associated *Pristionchus* species were also shown to form three or even five distinct mouth morphs (26). However, the dimorphism can also be lost during evolution, a process often referred to as ‘canalization’ (25). In this study, we describe a new example of a *Pristionchus* species with only one mouth form. Regardless of the intensive observation of the stomatal morphology using more than 2000 individuals of *P. coreanus* n. sp. from various conditions of cultures, e.g., different food bacteria and different age (population density), the eurystomatous form was not found during the observation. However, the stomatal shape, mostly width, varied among individuals, and individuals collected from starved culture (high population density) tended to have a relatively wide stoma with a clearly curved dorsal tooth, which is somewhat intermediate between the stenostomatous and eurystomatous forms, although their carnivorous behavior was not observed. These observations suggest that the eurystomatous form of *P. coreanus* n. sp. was lost or required a special condition for induction. Similar stomatal shape can be found in *P. uniformis* (2) and *P. seladoniae* (14). The eurystomatous form is rare in *P. uniformis*, and tentatively absent in *P. seladoniae*, and their stenostomatous form has clearly curved, large and an anteriorly directed dorsal tooth (2,14, Kanzaki, unpubl. obs.). Considering their apartness in the *Pristionchus* phylogeny (14) (Fig. 1), this character is likely to have occurred independently possibly according to their biological characters, e.g., preference of food and/or specified environment. In fact, *P. seladoniae* is hypothesized to inhabit a less competitive habitat, a subterranean bee nest where the necessity of the eurystomatous form is lower than in other habitats (14). Nevertheless, it might still be possible to induce the eurystomatous form of *P. coreanus* n. sp., and further culturing experiments are necessary to understand the feeding strategy of the species.

Regarding the molecular phylogeny, both new species fall into a subclade of the *pacificus* clade *sensu* (20). In 2015, this subclade included *P. pacificus*, *P. exspectatus*, *P. arcanus*, *P. maxplancki*, *P. japonicus* and *P. quartusdecimus*. Since then, nine new species were described, from Taiwan and China which expanded the *pacificus* clade *sensu stricto* and also formed a second clade around *P. quartusdecimus*. While the crown clade (*occultus* until *japonicus*) seems to be of Japanese/Taiwanese origin, the remainder has a Japanese/Chinese/Korean distribution. This fits well with the notion that the whole phylogeny of *Pristionchus* has a biogeographical pattern. The sister clade to this new *pacificus* clade *sensu lato* is a group of species that were collected in North and South America. The discovery of new *Pristionchus* species is still ongoing and seems far from saturation. With the systematic screen for new species, this project sets *Pristionchus* as an example for nematode biodiversity and we will see in the years to come how many more species this genus might include.
